# Establishment of ELISA method for canine adenovirus type 1

**DOI:** 10.3389/fvets.2024.1440124

**Published:** 2024-08-27

**Authors:** Ben Wang, Jinfeng Xu, Hui Zhang, Shizhen Lian, Yichang Duan, Hongling Zhang, Wei Hou, Baishuang Yin, Yanzhu Zhu

**Affiliations:** ^1^College of Animal Science and Technology, Jilin Agriculture Science and Technology University, Jilin City, China; ^2^Institute of Special Animal and Plant Sciences of Chinese Academy of Agricultural Sciences, Changchun, China; ^3^College of Animal Science and Technology, Heilongjiang Bayi Agricultural University, Daqing, China

**Keywords:** canine adenovirus type 1, ELISA method, sensitivity, specificity, coincidence rates

## Abstract

Canine adenovirus (CAdV) had a high prevalence in fox populations and induced fox encephalitis. No ELISA kits specifically for CAdV-1 antigen had been commercialized for foxes in China. It is crucial to develop a rapid and accurate ELISA method for detecting of CAdV-1. The monoclonal antibodies (mAbs, IgG1A) and HRP-labeled polyclonal antibodies (pAbs) were used to establish the ELISA method in this experiment. The results showed that the optimal concentration and coating time for the mAbs (IgG1A) were 2.15 μg/mL and overnight at 4°C, respectively. The dilution ratio of the HRP-labeled pAbs was 1:2000. Five percent skimmed milk was selected as the blocking agent. The optimal incubation times for blocking, CAdV-1, and HRP-labeled pAbs were all 1 h. The cut-off value for negative rectal swab was determined to be 0.366 ± 0.032. The maximum dilution ratio was 100 TCID_50_/mL. The ELISA method was positive to CAdV-1, and that was negative to CAdV-2, Canine Parvovirus (*CPV*) and Canine Distempervirus (*CDV*). The ELISA method showed good repeatability, sensitivity, and specificity. Compared with RT-PCR, the sensitivity, specificity, and coincidence rates of the ELISA method were 93.75, 90.9, and 92.86%, respectively. These results indicate that the established ELISA method can be used for the large-scale screening and epidemiology surveillance of CAdV-1 in foxes.

## 1 Introduction

Canine adenovirus type 1 (CAdV-1) is the aetiological agent of fox encephalitis (1). It is widely distributed geographically and evidence of infection has been found in foxes species worldwide. CAdV-1 was found in the fox of China (2), and the biological characterization of the CAdV-1 in the fox was clarified (3). The infection of CAdV-1 in the fox attract more attention. CAdV-1 was documented in a free-living hoary fox pup co-infected with canine distemper virus (CDV) in Brazil (4). One out of 52 foxes tested positive for CAdV was reported in free-ranging carnivores from Brazil (5). CAdV-seropositive red foxes and arctic foxes from Norway were found (6). Red foxes could be considered potential shedders of CAdV-1, as they showed a relatively high prevalence without related pathologic changes (7). Due to its adverse effects on foxes and the risk of dissemination to other countries, CAdV-1 in the fox attract more attention.

There are several methods for detecting canine adenovirus (CAV) antigen, including virus isolation, polymerase chain reaction (PCR), histopathology, rapid immunochromatographic strip assay and immunohistochemistry. Sequence and phylogenetic analyses were performed to identify the CAdV-1 in domestic dogs in southern Italy (8). The cytopathic effects, sequencing and electron microscopy were used to identify the CAdV-1 in the fox (2). The Immunochromatographic Strip test was found to be a sufficiently sensitive and specific detection method for the convenient and rapid detection of CAdV (9). The immunohistochemistry was used to confirm CAdV-1 infection on brain tissue of in a 5-week-old puppy (1). Conventional PCR was used to screen the CAdV-1 and CAdV-2 in the tissue samples from Raccoon dogs in Korea (2017–2020) (10). A reverse transcription polymerase chain reaction (RT-PCR) panel was desinged for detection and quantification of nine pathogens included CAdV-2 (11). The PCR/RT-PCR were used to detect circulating infectious agents including canine adenovirus in 1777 samples collected from Beagle dogs in China (12). The hexon gene-based PCR was used to detect the AdV in calves (13). RT-PCR and conventional PCR and/or sequencing were used to detect a total of 100 blood samples for carnivore protoparvovirus-1 (CPPV-1), CAdV-1/2, canine circovirus (CaCV), and CDV (14). Molecular techniques (PCR/qPCR) were often utilized to investigate the pathogen in the dogs and foxes. These methods have different detection drawbacks, such as long detection period and small-scale screening. It is essential to establish large-scale screening methods for CAdV-1 in foxes. However, limited ELISA method was used to detect the pathogen of CAdV-1. Thus, It is essential to build the large-scale screening ELISA methods for CAdV-1 in foxes, and It will be helpful to supervise the epidemiology of the CAdV-1.

## 2 Materials and methods

### 2.1 Ethics statement

The rectal swabs collection of the experimental procedures outlined below were reviewed, approved, and conducted in compliance with the guidelines of the IACUC of the Jilin Agriculture Science and Technology Univeristy and Institute of Special Animal and Plant Sciences of Chinese Academy of Agricultural Sciences. The approved research protocol number was LLSC202301010.

### 2.2 Horseradish peroxidase (HRP) labeling

The rabbit polyclonal antibodies (pAbs) were obtained by the member of our team in our previous research. The HRP Labeling Kit (Beijing bo’aolong Immune Technology Co., Ltd) was used to mark the rabbit pAbs with HRP (15). A 1/10 volume of the Marking buffer was added to the 2 mg/mL of the pAbs. The same mass of HRP was added to the labeled solution. The solution was mixed homogenously to avoid bubbles and was stored at room temperature for 3 h without light. Consequently, an appropriate amount of reaction termination solution was added it into the labeled reaction tube at a ratio of 1 μL for every 10 μL reaction solution. Next, It was mixed well and stored it at room temperature for 1 h. Lastly, an equal volume of the labeled preservation solution was added, mixed evenly, and stored at −20°C.

### 2.3 Screening for the best concentration of mice mAbs and rabbit HRP labeled pAbs

The mAbs (IgG1A) were obtained by the member of our team in our previous research. The mAbs were diluted to different concentrations of 17, 8.6, 4.3, 2.15, 1.075, 0.5375, and 0.26 μg/mL, and the HRP labeled pAbs were diluted to 1:500, 1:1000, 1:2000, 1:4000, and 1:8000 ratios. The mAbs with different gradients were coated with plates and kept at 4°C overnight. Three washes with PBST were performed and 5% skimmed milk was added and blocked for 2 h. The CAdV-1 was incubated at 37°C for 1 h. Subsequently, three washes using PBST were performed. The HRP labeled pAbs with different dilutions were incubated at 37°C for 1 h. The TMB solution was used for 15 min. Lastly, after sulfate termination, the OD value was detected at 450 nm using an enzyme-labeled instrument (Bio-Rad, USA). The P/N value was calculated by the positive and negative OD value. The higher P/N value indicates the best situation of the ELISA.

### 2.4 Optimized ELISA method

The antibody coating concentrations were coated in the plate. The coating times were 2 h, 3 h, 4 h, and overnight at 4°C. Three washes with PBST were performed. Three percent BSA, 1% gelatin, 1% casein, and 5% skimmed milk were selected as the blocking solutions and they were incubated at 37°C for periods of 0.5, 1, 1.5, and 2 h. The CAdV-1 was incubated at 37°C for 0.5, 1, 1.5, and 2 h. The concentration of HRP-labeled pAbs were added, and their incubation time was 0.5, 1, 1.5, and 2 h at 37°C. The TMB solution was used to react for 15 min. Following termination with sulfuric acid, the optical density (OD) was measured at 450 nm using an enzyme-labeled instrument (Bio-Rad, USA) (16). Thus, the ELISA method condition was optimized.

### 2.5 The cut-off value

According to the determined optimal reaction conditions of ELISA, the cut-off value of 30 negative rectal swabs were determined (17). Three replicate wells were done for each rectal swabs, the OD 450 value was read, the mean and standard deviation (SD) were calculated, and mean + 3SD was used as the threshold value. When detection value was higher than the cut-off value, the rectal swabs can be judged as positive; when detection value was lower than the cut-off value, the rectal swabs can be judged as negative.

### 2.6 Repeatability test

The same batch of purified mAbs coated plates was used in the inter-plate repeatability test. The plates coated with purified mAbs from different batches were used in the intra-plate repeatability test. Under the optimized experimental conditions, 10 positive and two negative samples were detected. Notably, three parallel controls were prepared for each sample. Coefficient of variation (CV) = (SD/mean) × 100%.

### 2.7 Sensitivity test

The TCID_50_ of the CAdV-1 was 10^8^/mL. The different CAdV-1 dilution ratios (10^−7^, 10^−6^, 10^−5^, 10^−4^, 10^−3^, 10^−2^, and 10^−1^ TCID_50_) were determined using the optimized ELISA test. Finally, three replicates were set for each dilution sample, and the positive and negative controls were also set for ELISA detection.

### 2.8 Specificity test

CAdV-1, CAdV-2, CDV, and canine parvovirus (CPV) were determined through the optimized ELISA method. Three replicates were set for each sample, and the OD value was detected at 450 nm using an enzyme-labeled instrument (Bio-Rad, USA).

### 2.9 Comparison of the ELISA method and RT-PCR

The rectal swabs were collected from the fox farm in Jilin and Heilongjiang Provicne, and were stored at –20°. They were placed in sterile test tubes containing sterilized saline, left to stand for 5–10 min, shaken for 1 min, the rectal swabs were removed, centrifuged at 10,000 rpm for 10 min, the supernatant was collected and preserved at 4°C for the next step in the assay. There were 48 CAdV-1 positive and 22 CAdV-1 negative rectal swabs in our Lab. RT-PCR (18) and optimized ELISA were used to detect 70 blind rectal swabs in our laboratory. Compared with the RT-PCR, the specificity, sensitivity, and coincidence rate of the antigen ELISA method were calculated, respectively.

### 2.10 Statistical analysis

**Figure 1:**
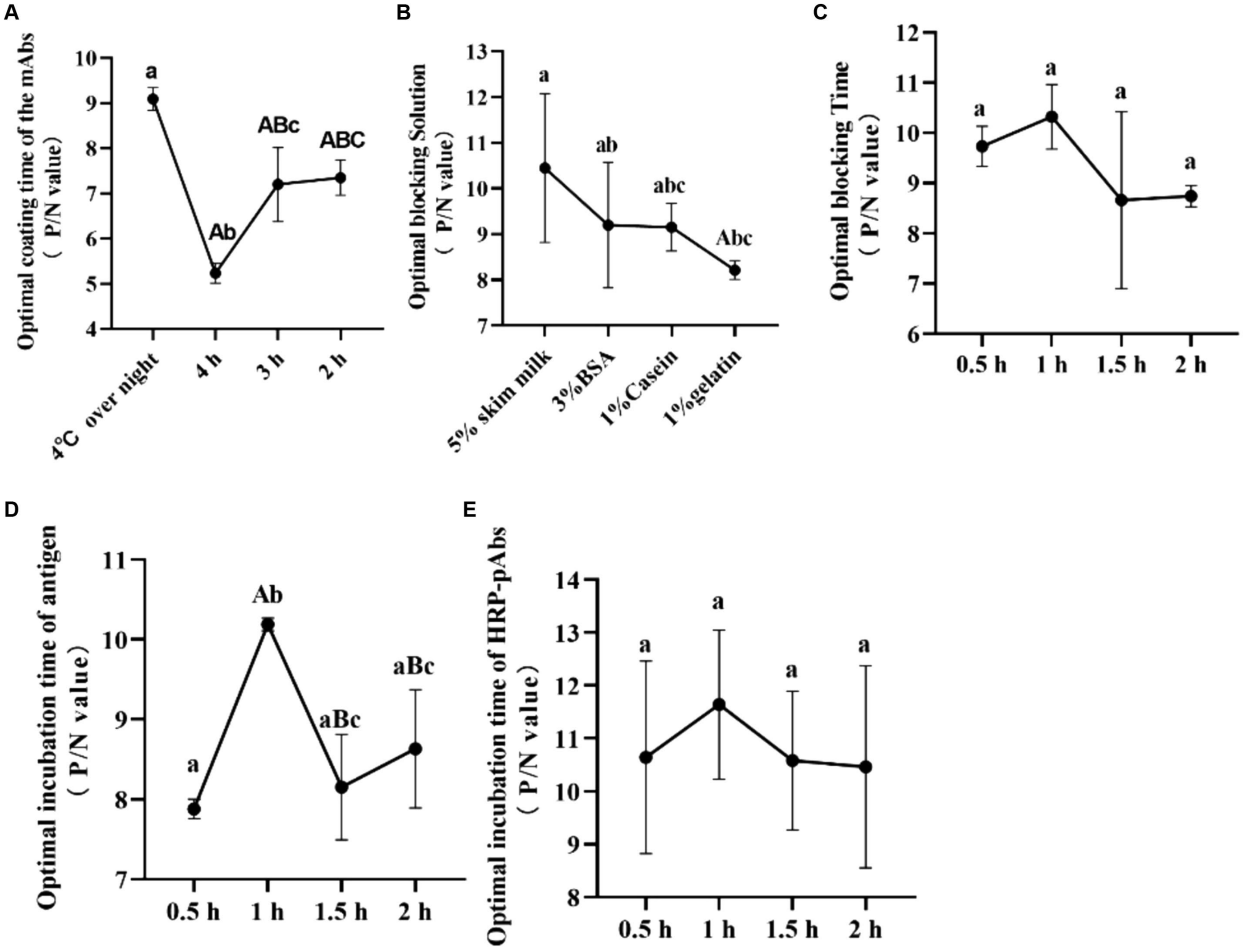
The optimal procedure of the double antibody sandwich ELISA. **(A)** The optimal coating time of the mAbs (IgG1A); **(B)** the optimal blocking solution; **(C)** the optimal blocking time; **(D)** the optimal incubation time of antigen; **(E)** the optimal incubation time of HRP-pAbs. The same letter was considered not significant, while differences with different letters of the same sign are considered significant.

Three replicates were set for each rectal swab. In Figure 1, statistical analyses of the optimal procedure of the ELISA were done using SPSS 22.0 for Windows (SPSS Inc., Chicago, IL, USA) with one-way analysis of variance followed by Student’s t-test. The Shapiro–Wilk test was used to verify the normal distribution of data, and not-parametric Kruskal–Wallis and Mann–Whitney tests were also used. The same letter was considered not significant, while differences with different letters of the same sign were considered significant. The P/N value was calculated from the Tables 1–5 by the ratio of the positive and negative OD value. The higher P/N value indicates the best situation of the ELISA. The OD value of the positive rectal swab was higher than the cut-off value. The data were expressed as means ± standard deviation.

**Table 1:** Optimum working concentration of antibodies.

mAbs	HRP-PAbs	1:500	1:1000	1:2000	1:4000	1:8000
17 μg/mL	P/N	3.713 ± 0.156	3.468 ± 0.212	3.629 ± 0.02	3.768 ± 0.02	3.71 ± 0.056
8.6 μg/mL	P/N	3.514 ± 0.031	3.239 ± 0.137	3.733 ± 0.032	3.668 ± 0.08	3.696 ± 0.077
4.3 μg/mL	P/N	2.868 ± 0.042	2.191 ± 0.222	3.302 ± 0.12	3.659 ± 0.027	3.387 ± 0.061
2.15 μg/mL	P/N	3.594 ± 0.043	3.584 ± 0.134	4.219 ± 0.046	3.59 ± 0.056	3.623 ± 0.084
1.075 μg/mL	P/N	3.37 ± 0.07	3.307 ± 0.051	3.35 ± 0.092	3.316 ± 0.038	3.229 ± 0.13
0.5375 μg/mL	P/N	3.069 ± 0.115	3.048 ± 0.273	3.052 ± 0.247	3.046 ± 0.177	2.997 ± 0.246
0.26 μg/mL	P/N	2.363 ± 0.021	2.335 ± 0.167	2.588 ± 0.042	2.643 ± 0.136	2.471 ± 0.288

**Table 2:** Repeatability of ELISA method.

Item	The intra-assay repeatability		The inter-assay repeatability	
	CAdV-1	Negative control	CAdV-1	Negative Control
1	1.885 ± 0.23	0.21 ± 0.05	2.25 ± 0.23	0.22 ± 0.07
2	1.832 ± 0.07	0.21 ± 0.04	2.33 ± 0.15	0.24 ± 0.03
3	2.064 ± 0.12	0.24 ± 0.09	2.41 ± 0.17	0.23 ± 0.06
4	1.736 ± 017	0.24 ± 0.08	2.47 ± 0.15	0.24 ± 0.03
5	1.723 ± 0.07	0.23 ± 0.05	2.23 ± 0.1	0.23 ± 0.03
Variable coefficient (CV%)	7.48%	6.71%	4.39%	3.61%

**Table 3:** Sensitivity of ELISA method.

CAdV-1 (TCID_50_/mL)	10^−1^	10^−2^	10^−3^	10^−4^	10^−5^	10^−6^	10^−7^
OD450	3.049 ± 0.21	2.523 ± 0.31	1.98 ± 0.12	1.176 ± 0.25	0.726 ± 0.08	0.421 ± 0.03	0.246 ± 0.06

**Table 4:** Specificity of the ELISA method.

Antigen	OD450		P/N value
CAdV-1	2.31 ± 0.26	0.23 ± 0.05	10.25 ± 1.8
CAdV-2	0.31 ± 0.05	0.28 ± 0.03	1.11 ± 0.07
CDV	0.27 ± 0.07	0.31 ± 0.06	0.88 ± 0.18
CPV	0.24 ± 0.02	0.26 ± 0.02	0.92 ± 0.04
Negative	0.25 ± 0.04		/

**Table 5:** Coincidence rate between ELISA and RT-PCR method.

Methods	RT-PCR positive	RT-PCR negative	Sum
ELISA positive	45	2	47
ELISA negative	3	20	23
Sum	48	22	70
Sensitivity	93.75%		
Specificity		90.9%	
Co-incidence			92.86%

## 3 Results

### 3.1 Optimal dilution concentration of mAbs and HRP labeled pAbs

Utilizing the chessboard titration method, the optimal coating concentration for mAbs was determined to be 2.15 μg/mL. In contrast, for the horseradish peroxidase (HRP) labeled rabbit pAbs, the dilution ratio was identified as 1:2000, yielding a positive-to-negative (P/N) ratio of 4.219 ± 0.046, indicating an optimal condition for the assay (Table 1).

### 3.2 The optimal coating conditions

The mAbs exhibited a higher positive-to-negative (P/N) value when coated condition was overnight at 4°C, outperforming those coated for durations of 4, 3, and 2 h. Consequently, the optimal coating condition for the mAbs was established as an overnight period at 4°C, providing the most favorable results (Figure 1A).

### 3.3 The blocking fluid and time

5% skimmed milk, 3% BSA, 1% Casein, and 1% Gelatin were screened to find the best blocking fluid. The P/N value of the detected sample was highest when 5% skimmed milk was used for blocking. Thus, 5% skimmed milk produces the best-blocking effect (Figure 1B). And 0.5, 1, 1.5 and 2 h were used to screen the best blocking time. The P/N value of the detected sample was highest when the time was 1 h. Thus, the best-blocking time was deemed to be 1 h (Figure 1C).

### 3.4 The CAdV-1 incubation time

The incubation time of CAdV-1 was screened, and 0.5, 1, 1.5 and 2 h were used to screen. The highest P/N value of the detected sample was highest when 1 h was used for incubation, and the significant difference was observed. It indicates that the optimal incubation time was 1 h (Figure 1D).

### 3.5 Screening of the HRP labeled PAbs condition

The incubation time of the HRP labeled PAbs was screened, and 0.5, 1, 1.5 and 2 h were used to screen. The highest P/N value of the detected sample was highest when 1 h was used for incubation. Therefore, the optimal incubation time of the HRP labeled pAbs was 1 h (Figure 1E).

### 3.6 The cut-off value

The cut-off value was used as threshold value to distinguish between positive and negative results in the ELISA method. The cut-off value of the negative rectal swabs was 0.366 ± 0.032. The sample was positive when its OD value was higher than the cut-off value. The sample was negative when its OD value was lower than the cut-off value.

### 3.7 Repeatability tests

In the inter-batch repeatability test, the coefficient of variation of the sample was 7.48 and 6.71%, respectively. The coefficients of variation were less than 10%, with good inter-assay repeatability. In the Intra-assay repeatability test, the coefficient of variation of the sample was 4.39 and 3.61%, respectively. The coefficients of variation were less than 5%, with good intra-batch repeatability (Table 2). It indicates that the ELISA method had good repeatability.

### 3.8 Sensitivity test

The TCID_50_ of the CAdV-1 was 10^8^ /mL. Seven doses of the CAdV-1 was used to determine the sensitivity of the ELISA method. The OD450 value of the 10^−6^ was higher than the cut-off value, and It indicates that the maximum dilution ratio was 100 TCID_50_/mL (Table 3).

### 3.9 Specificity test

CAdV-1, CAdV-2, CPV and CDV were used to determine the specificity of the ELISA method. The OD450 value of the CAdV-1 was higher than the cut-off value, and indicates that the detection result was positive. The OD450 values of the CAdV-2, CPV and CDV were lower than the cut-off value, and indicates that the detection result was negative (Table 4). The P/N value of the CAdV-1 was highest. Thus, the ELISA method was specified for the CAdV-1.

### 3.10 Comparison of ELISA and RT-PCR

Seventy clinical samples, which were collected from fox farms in the Jilin and Heilongjiang Province, were stored in our Lab. In the 70 samples, 47 were positive and 23 were negative for CAdV-1 after applying the ELISA, whereas 48 were positive and 22 were negative after using RT-PCR. The sensitivity, specificity, and coincidence rates were 93.75, 90.9, and 92.86%, respectively (Table 5).

## 4 Discussion

Infectious canine hepatitis is caused by CAdV-1, a double-stranded naked DNA virus belonging to the family *Adenoviridae*, genus *Mastadenovirus*. The infection mainly affects dogs and other carnivores, and damages the kidneys, liver, and eyes. Early diagnosis of CAdV-1 is crucial for clinical treatment, but the non-specific nature of the infection makes its early diagnosis difficult, resulting in the enlargement infection of CAdV-1 in fox. ELISA is widely used in the diagnosis of infectious diseases and antibody detection because of its simplicity, low requirements for laboratory equipment and high sensitivity. The ELISA method has a higher specificity and better sensitivity concerning indirect assays (19). The majority of dogs had antibody levels against CAdV (20, 21). Thus, CAdV-1 pathogen ELISA method is expected to be translated into a routine kit for large-scale screening and prognostic assessment.

The sensitivity and specificity of ELISA assays are affected by a number of factors in the antigen–antibody reaction (22). In the antigen–antibody reaction, if there is an excess of antibody or antigen, the formation of antigen–antibody complexes is reduced, and the amount of antigen to be measured is lower than the actual amount, resulting in a high false-negative rate, so it is necessary to determine the optimal working concentration of the capture antibody and detection antibody. Therefore, it is necessary to determine the optimal working concentration of the capture antibody and detection antibody. The mAbs (IgG1A) and pAbs have been identified to be specific to CAdV-1, which increases the specificity of the antigen ELISA method kit. According to the chessboard method, the optimal coating concentration of mouse mAbs was 2.15 μg/mL, whereas the dilution ratio of HRP labeled rabbit pAbs was 1:2000. After repeated screenings, an pathogen ELISA method kit was constructed, in which mAbs (IgG1A) and pAbs had been employed as the capture antibody and detection antibody. As the detection antibodies in ELISA method, pAbs can efficiently increase the sensitivity by nearly 10-fold (23). The two antibodies can bind to multiple epitopes of the antigen, reducing the missed detection rate (24).

The coating concentration and time, sample reaction condition, blocking time, as well as the HRP-labeled antibody dilution and reaction condition are essential to screen during the establishment of ELISA (17). In this study, the ELISA reagents and incubation conditions were sellected as the initial reaction system, and then optimized each step or condition to minimize the effect of non-specific binding, so as to determine the optimal conditions for the ELISA. The optimized procedure improves and guarantees the sensitivity of the ELISA method. Specificity is also important to the pathogen ELISA (25). In this experiment, the ELISA method was not specific to the CAdV-2, CPV and CDV. The sensitivity is essential to the ELISA Kit (26). In our study, the sensitivity of the ELISA method was up to 100 TCID_50_/mL CAdV-1.

A reproducibility test that provides the coefficients of variation when analyzing sera from intra-plate and inter-plate is necessary (27). Precision is an assessment of the consistency of repeated test results, the lower the coefficient of variation (CV), the higher the precision of the assay. Accuracy is an assessment of the consistency between the test value and the theoretical value, a recovery rate that is closer to 100% indicates superior accuracy in the measurement process. According to national regulations, commercial ELISA kits should have a CV of less than 10% and a recovery rate of 80–120% (28). In our instance, the intra-and inter-assay repeatability was 7.48 and 4.39%. The ELISA parameters established in this study were all within the reference range, indicating good precision and accuracy. It speculates that it is a good level of reproducibility for the ELISA method.

Determining the coincidence rate of the designed method was crucial. RT-PCR had already been established and was currently used to determine the CAdV-1 (29, 30). But limited data showed the pathogen ELISA for CAdV-1. Compared with RT-PCR, the ELISA method was helpful for large-scale screening of samples. The pathogen ELISA established in this study can detect CAdV-1 in rectal swabs, and has potential application in the clinical antigen detection of CAdV-1. However, the ELISA method also has its pitfalls. In particular, ELISA-based tests often have difficulty detecting pathogen during the initial phase of infection, which necessitates the collection of samples during the middle to late stages of infection.

## 5 Conclusion

The pathogen ELISA established in this study can detect CAdV-1 in rectal swabs, and has potential application in the clinical antigen detection of CAdV-1. It can be used for both practically and financially unfeasible to attempt a disease eradication program for CAdV-1 in red foxes. In the future, This ELISA method will be used to establish large-scale screening for CAdV-1 in dog and foxes.

## Data Availability

The datasets presented in this study can be found in online repositories. The names of the repository/repositories and accession number(s) can be found in the article/supplementary material.
